# Management Strategies for Atrioventricular Groove Rupture After Mitral Valve Surgery

**DOI:** 10.1016/j.jaccas.2026.109909

**Published:** 2026-09-02

**Authors:** Muhammad Junaid Mustafa

The case report presented by Cristiano et al^1^, provides valuable insights that compel us to read it with great interest. Left atrial dissection during mitral valve surgery^2^ is an extremely rare but devastating complication. Because redo surgery^2^ in the context of an atrioventricular groove rupture carries an exceedingly high risk of massive hemorrhage and mortality, clinicians are often left with difficult choices. The authors should be commended for saving a 60-year-old woman by making these bold clinical decisions.

This procedure highlights 2 valuable clinical lessons that warrant further emphasis:

As the investigators noted, the patient initially demonstrated hemodynamic stability. However, serial 3-dimensional transesophageal echocardiography revealed a rapidly expanding false lumen (FL) and early left ventricular inflow obstruction. This demonstrates that early hemodynamic stability can be deceptive, underscores the necessity of mandatory surveillance, and reminds us not to rely on transient clinical stability.

## Anatomical Precision via a Multimodality Approach

This case report illustrates the critical importance of anatomical precision and demonstrates why a multimodality imaging approach is essential. Passing a guidewire through a femoral approach, retrogradely descending it via the aortic valve into the left ventricle, and subsequently navigating it through the rupture into the FL requires extreme precision. While transesophageal echocardiography played a vital role, cardiac computed tomography provided the exact localization of the FL and rupture. By accurately mapping the serpiginous tunnel and its proximity to the coronary sinus and right coronary artery, the team safely selected a muscular ventricular septal defect occluder with a 7-mm waist. This approach led to the complete closure of the FL without complications.

Furthermore, the investigators' intraprocedural problem-solving was commendable. Realizing that the FL was retrogradely refilling from the right atrium, they immediately took steps to close the residual interatrial septal fenestration using an atrial septal defect occluder. This proactive management led to a favorable prognosis, with no residual defect observed at the 6-month follow-up.

In conclusion, Cristiano and his team have laid an excellent foundation for managing such complex cases. They have provided the Heart Team with a vital, lifesaving blueprint when facing a lethal postsurgical complication in patients with high-risk.

## References

[1] Cristiano M., Santoro G., Romano A. (2026). Percutaneous closure of an atrioventricular groove rupture causing a left atrial dissection after cardiac surgery. JACC Case Rep.

[2] Alshaabi H., Donaghue J.F., Franko D.M., McCullough J.N. (2024). Recognition and management of left atrial dissection during mitral repair. J Cardiothorac Surg.

